# Proceedings from the CIH^LMU^ 5th Infectious Diseases Symposium 2016 “Drug Resistant Tuberculosis: Old Disease - New Challenge”

**DOI:** 10.1186/s12919-017-0077-6

**Published:** 2017-09-04

**Authors:** Celso Khosa, Krutarth Patel, Karlygash Abdiyeva, Nurkeldi Turebekov, Bettina Prüller, Norbert Heinrich

**Affiliations:** 1 Center for International Health – CIHLMU, Munich, Germany; 2 Instituto Nacional de Saúde, Maputo, Moçambique; 3 Alere Technologies GmbH, Jena, Germany; 4 Kazakh National Medical University, Almaty, Kazakhstan; 5 Division of Infectious Diseases and Tropical Medicine, Medical Center of the University of Munich, Munich, Germany; 6 German Center for Infection Research (DZIF), Munich partner site, Munich, Germany

**Keywords:** Multi-drug resistant tuberculosis, Extensively drug-resistant tuberculosis, Diagnosis, Therapy

## Abstract

The 5th CIH^LMU^ Infectious Disease Symposium, Munich, Germany, March 12, 2016 brought together Tuberculosis Experts from developed and low middle-income countries to discuss the control of drug resistance Tuberculosis. The meeting featured 9 presentations: Tuberculosis history and current scenario, Tuberculosis and migration - current scenario in Germany, Mechanism of Tuberculosis resistance development, Epidemiology of resistance – transmission vs. new generation of resistance, The impact of diagnostic in patients beyond – sensitivity and specificity, The Bangladesh regimen – new hope trough old drugs, New drugs and regimens – an overview on studies and Multi and Extensively Drug Resistant Tuberculosis from Europe. The presentations were followed by a panel discussion.

Serious Multidrug Resistance epidemic in some countries may jeopardize the progress in Tuberculosis control. In this meeting epidemiology, mechanism, immigration and screening, diagnosis, research and treatment of drug resistant tuberculosis were discussed.

## Introduction

**Fig. 1:**
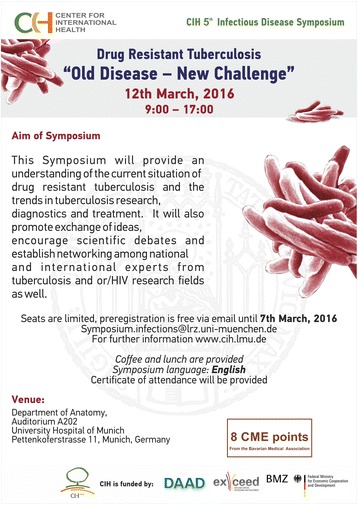
Symposium flyer

The Center for International Health – CIH^LMU^ convened the 5th CIH Infectious Disease Symposium – Drug Resistant Tuberculosis “Old Disease – New Challenge” on the 12th of March 2016, in Munich, Germany (Fig. 1). Tuberculosis (TB) is an old disease, but not a disease from the past, TB is still a public health challenge in many countries. In 2014, 9.6 million cases of TB were estimated worldwide, 3.3% of new cases and 20% of previously treated cases had Multidrug Resistant TB (MDR-TB) [1].

The aim of this symposium was to discuss holistically the drug resistant TB in developing and developed settings. Experts with diverse background and specialties were invited to discuss the epidemiology, etiology, diagnosis, prevention and treatment of drug resistant TB in different settings.

## Presentation summaries

### Tuberculosis history and current scenario

Dr. Nilesh Bhatt, Instituto Nacional de Saúde, Maputo, Mozambique.

### Summary of presentation

*Mycobacterium ulcerans* existed since the Jurassic period (150 million years ago) [2], *Mycobacterium tuberculosis* (MTB) emerged about 3 million years ago [2] and was first described by Robert Koch in 1882. MTB is a gram-positive rod, acid fast with a slow generation time and non-replicant persisters (latency, hard to kill). MTB is airborne transmitted by droplet and the main epidemiological important form is the pulmonary tuberculosis. In pre-chemotherapy era (before 1940) bed rest and sunlight and in certain cases, surgery - were the only treatment available.

The first case of MTB resistance to Streptomycin was described in 1948 [3] Resistance to TB antibiotics are classified as sensible, mono-resistance, MDR and Extensively Drug-resistant (XDR). The degree of resistance is directly associated with duration, cost and toxicities of the treatment. The proportion of new cases with MDR-TB remains unchanged. However, serious MDR-TB epidemics in some countries jeopardize progress in TB control. There are gaps in resistance testing versus the use of new technologies. Only 50% patients have MDR TB and only a quarter completes XDR TB treatment [1].

## Discussion

- Diagnosis and treatment gaps are still a challenge to control drug resistant TB worldwide;
- Current drug resistant TB treatment is lengthy, toxic and expensive;
- New, safer drugs and regimes are urgently needed to improve treatment outcomes for drug resistant TB.

### Tuberculosis and migration-current scenario in Germany

Dr. Lena Fiebig, Robert Koch Institute, Berlin, Germany.

### Summary of presentation

Tuberculosis rates in Germany have been declining in the second half of the twentieth century and beginning of the twenty-first century. Nevertheless, there has been an increase in the tuberculosis notification rates from 5.6/100 000 to 7.3/100 000. In general, this can have several reasons: 1) ongoing domestic spread of tuberculosis in Germany; 2) reactivation of Latent Tuberculosis Infection (LTBI) in the native population who has acquired the infection in the past; or 3) reactivation of LTBI in inhabitants who have acquired the infection in another country of origin; as well as 4) entry of individuals into the country with existing (diagnosed or undiagnosed) active TB. These scenarios require different actions in TB prevention and control [4].

Available data indicate that the increasing TB case numbers are influenced by a changing demographic context and migration. An increasing number of cases have been identified in the context of active case finding (screening) among asylum seekers and refugees according to the Protection Against Infection Act (Infektionsschutzgesetz).

## Discussion

- Tuberculosis remains a public health challenge in Germany, the declining of TB trend in Germany has come to an end;
- Tuberculosis cases in Germany are increasing in the context of migration, concerted action in all levels of TB control is key, active case finding, including both screening and contact tracing, gains importance;
- Drug resistant TB gains importance. The proportion of MDR-TB was highest in patients born in one of the Newly Independent States (NIS) and among patients who have previously received anti-TB treatment;
- Need for better diagnostic tools for screening, chest X-ray screening is not perfect but an appropriate initial test for detecting pulmonary Tuberculosis;
- Treatment success was 77% for cases notified in 2014 and did not reached World Health Organization (WHO) targets of 85% [5].

### Mechanism of tuberculosis resistance development

Dr. Norbert Heinrich, Ludwig-Maximillians-Universität, Munich, Germany.

### Summary of presentation

Spontaneous drug resistance mutations occur at likelihood per cell division of 10^−6^ for Isoniazid (INH), 10^−8^ for Rifampicin (RMP). Selection of resistant bacteria can happen by inadequate drug combinations or by monotherapy. The use of multiple drugs in Fixed Dose Combinations (FDC) is expected to prevent newly acquired drug resistance.

The classical hypothesis is that patient factors were responsible for the appearance of resistance. Those were poor adherence partly due to long treatment, which would make patients stop their treatment early. However, more recent pre-clinical studies using the hollow fiber model [6] simulated plasma drug concentrations to replicate non-adherence show that non-adherence does not allow a drug-resistant subpopulation to overgrow. A study in Cape Town with 10 000 patients simulated on 6-month treatment with 100% adherence, assumed pharmacokinetic (PK) variability factored into the simulation, 0.68% of patients developed MDR TB despite 100% adherence [7]. This was further confirmed by a meta-analysis of clinical studies involving 6510 patients, which revealed that INH rapid acetylators who have lower drug concentrations, had a Risk Ratio (RR) of 2.0; 95% confidence interval [CI], (1.5–2.7) for microbiological failure and RR of 2.0 (1.1–3.4) for acquired drug resistance [8].

Heat maps of drug distributions into the TB lesions show that a difference in penetration of drugs into the most difficult lesion component, the necrotic component. In current first line therapy, this leads to RMP most likely being the only drug which penetrates the necrosis and is active on bacilli encountered in there. New drugs in development often do not penetrate the necrotic portion of the lesion in high concentrations [9].

## Discussion

- Current data is not complete regarding the mechanism of resistance development;
- Classical hypothesis (no adherence) increasingly replaced by PK variability/local monotherapy hypothesis;
- Insufficient concentrations of main and companion drugs;
- Mechanisms likely include: PK variability, especially in critical lesion compartments pathogen related factors and no adherence;
- Optimize existing and new combinations for dosing in the entire population, by animal PK/ Pharmacodynamics (PD)/lesion studies paired with human PK/PD and population modelling;
- Pharmacogenomics; tailor the doses to acetylator status improving outcomes in terms of treatment success and less toxicity;
- INH prophylactic treatment and resistance; usually does not lead to resistance because of low number of microorganism.

### Epidemiology of resistance – Transmission vs. new generation of resistance

Dr. Matthias Merker, Research Center, Borstel, Germany.

### Summary of presentation

There are approximately 500 000 new MDR-TB cases each year, and 200 000 patients die from that form of TB. Only 1 out of 3 estimated cases is diagnosed (gap in diagnosis) and treatment success rate is low globally with 48% (gap in treatment) suggesting high rates of MDR-TB transmission in the community and/or hospital. The city of Minsk, Belarus recently reported every second new TB patient to be infected with MDR-TB strains [10]. Also other MDR-TB high burden settings, especially countries of the former Soviet Union, are confronted with high treatment costs of M/XDR-TB cases of over 100 000 dollar per patient as well as severe side effects of second-line drugs, leading to low cure rates comparable to the pre-chemotherapy era in some settings [1].

As one paramount example for increasing MDR-TB transmission rates Karakalpakstan, a region in Uzbekistan suffers from an increase of new MDR-TB cases from 13% in 2001 to 26% in 2012 despite the subsequent introduction of WHO endorsed Directly Observed Treatment, Short-course (DOTS) strategy in 1998, and extended second-line treatment program in 2003. Two MDR cohorts were followed from 2001 to 2006 based on standard genotyping showing that the MDR-TB strain population is dominated by strains belonging to the Beijing genotype.

## Discussion

- Treatment programs need to consider/trace dominating strain populations and resistance levels carefully;
- There is a need of early and correct detection of resistance profiles;
- Risk for further resistance development in failing MDR-TB treatment regimens.

### The impact of diagnostic in patients beyond – Sensitivity and specificity

Dr. Katharina Kranzer, Research Center, Borstel, Germany.

### Summary of presentation

The sensitivity of diagnostic tests depends on the bacterial load in a sample. Limits of detection are 1–50 cfu/ml for Mycobacterial culture, 130 cfu/ml for Nucleic Acid Test (NAT) (eg. Xpert MTB/RIF) and 5000–10 000 cfu/ml for smear microscopy. Sensitivity of test depends on the distribution of the bacterial load within individuals with TB. Cost, feasibility, safety, accuracy, reproducibility, health system, treatment and comorbidities need to be taken in to consideration when evaluating new diagnostics. Accuracy studies suffice if a new diagnostic test is safer or more specific but of similar sensitivity to the old test. However, if a new test is more sensitive it will lead to additional cases being detected. Results of clinical trials assessing treatment efficacy enrolling patients diagnosed by the old test may not apply to these additional cases. Randomized control trials (individual or cluster randomization) investigating the effect of new diagnostics on clinics outcomes might be at risk of confounding and might be context-specific, decreasing their generalizability.

Education and training of clinicians is important to ensure correct use and interpretation of new diagnostics. Quality management of diagnostic tests is important both within and outside of laboratories to ensure the correct result is issued for the right patient within a reasonable time frame.

## Discussion

- Sensitivity of diagnostic tests depends on limits of detection and the bacterial burden within a population;
- Evaluation of diagnostics tests with improved sensitivity should include clinical outcomes;
- Randomized controlled trials assessing the effect of diagnostics are prone to confounding and generalization might be limited;
- Quality control is widely advocated, but robust impact evaluation with regards to patient outcomes and cost-effectiveness are missing.

### The Bangladesh regimen – New hope through old drugs

Prof. Hans L. Rieder, University of Zurich and Tuberculosis consultant, Zurich, Switzerland.

### Summary of presentation

Starting in 1997, with recruitment into sequentially adaptive regimens for MDR-TB, carefully observing what happens to the patient bacteriologically and regarding treatment outcome, 6 sequentially adaptive regimens were used until obtaining the final 9-month regimen in 2002, composed of 7 drugs:

- 3 first-line drugs: INH, Ethambutol and Pyrazinamide;
- 1 Injectable: Kanamycin;
- 1 Anti-leprosy drug: Clofazimine;
- 1 Thioamide: Prothionamide;
- 1 Fourth-generation fluoroquinolone: Gatifloxacin;

The four-month intensive phase was prolonged if smear positive after 4 months (up to a maximum of 6 months; if then still positive, patient was declared to have treatment failure) and a fixed 5-month continuation phase, treatment duration between 9 and 11 months. The results were excellent Patients with a fluorquinolone-susceptible strain had a successful, relapse-free outcome in 87.7% (*n* = 439: death = 24, default = 31, failure = 1 and relapse = 1). Similarly, among patients with a low level of fluorquinolone resistance, the outcome was successful in 90.4% (*n* = 33: death = 0, default = 2, failure = 0 and relapse = 1) [11].

Another observational implementation study is ongoing in 9 francophone countries (Union-Africa collaborative study) with a preliminary success of over 80% [12].

## Discussion

- A short, effective treatment regimen for MDR-TB based on the core drug Gatifloxacin has been developed since 1997 in Bangladeshi patients;
- The regimen is composed of 7 generically available (including 3 first-line) drugs, plus an anti-leprosy drug;
- Synergy between the drugs might be the key for this regimen.

### New drugs and regimens – An overview on studies

Nyanda Elias Ntinginya, MD, MSc, PhD Candidate, NIMR Mbeya Medical Research Centre, Mbeya, Tanzania.

### Summary of presentation

TB is an old disease, but not a disease of the past. Challenges of current TB therapy:

- Old – Arsenal of drugs developed about 5 decades ago
- Long – TB treatment takes up to 30 months for drug resistant TB
- Complex – Pill burden oral + daily injections. Enhanced lab testing and hospitalization
- Expensive – Drug resistance TB therapy can cost US$ 5000–10 000
- Inadequate – MDR treatment often fails, lower success rates

There is an urgent need to increase access to effective, affordable and acceptable treatment for MDR-TB (Table 1).

**Table 1:** Anti TB drugs under evaluation in MDR/XDR clinical trials

Bedaquiline (Sirturo®; TMC207)	Diarylquinoline, blocking ATP synthetase, adaptive Licensing FDA: marketing approval for MDR end 2012. WHO interim recommendation by June 2013. By 2014, 43 countries had used, serious concern about QT-Prolongation [14]
Delamanid (OPC 67683)	Nitroimidazooxazole, Phase 2 results promising, Phase 3 ongoing (MDR). European Medicines Agency Committee for Medicinal Products for Human Use recommended licensing in Nov 2013. Safety: QT-Prolongation, WHO recommendation: Oct 2014 [15]
Fluoroquinolones: Moxifloxacin, Gatifloxacin	Class of broad-spectrum antibiotics that inhibit the DNA gyrase enzyme, effective in treating MDR-TB. Excellent early bactericidal activity, Lack sterilizing activity. Safety: QT prolongation, safety in combination therapy need further evaluation. Resistance and cross-resistance are reported and pose a threat. The optimal dose of Moxifloxacin and Levofloxacin – has not yet been ascertained. [16]
Pretomanid (PA-824)	Nitroimidazole, significant bactericidal and sterilizing activity alone and in combination tested in multiple MDR-TB regimens including the NC005, STAND and Nix-TB studies by TB Alliance MDR-TB treatment trial by MSF, the TB-PRACTECAL study, [16]
Clofazimine (CFZ)	Approved for leprosy treatment and used off-label for MDR-TB, tested in several MDR-TB regimens designed to shorten treatment Including the STREAM, TB PRACTECAL and end TB trials. Further studies underway [16]

## Discussion

- New potential drug candidates with novel regimen have entered clinical trials in recent years with one phase III trial ongoing;
- Studies on MDR-TB have their challenges and complexities that merit further exploration;
- Urgent need to increase access to effective, affordable and acceptable treatment for MDR-TB;
- Continued international collaboration among stakeholders is needed.

### MDR/XDR TB tuberculosis from Europe

Prof. Martin Boeree, Radboud University Medical Center, Nijmegen, Netherlands.

### Summary of presentation

After development of RMP, for 40 years no new TB drugs were introduced. TB incidence decreases in developed world while after an initial decrease it increases in limited resources setting. TB incidence increases relate to HIV, emergence of drug resistance and poverty.

Guidelines from WHO and in different countries especially for the treatment of drug resistant TB are based on poor evidence.

MDR and XDR-TB can be cured if at least 4 s line drugs, 8 months intensive phase with the use of an injectable. Uncertainty about optimal dose, duration and combinations are factors affecting treatment success. If drugs and rapid diagnostic would be available, individualized treatment is affordable, low resource countries need a programmatic approach. Serious side effect occurs in up to half of the patients [13].

Use of older drug, repurposed drugs and host directed therapies as means to development of new regimens for the treatment of MDR-TB. New drug like Bedaquline and Delamanid should be used in adequately structured regimens to avoid development of resistance to those drug, avoiding to repeat errors from the past.

## Discussion

- Programmatic versus individualized approaches to MDR-TB treatment;
- Use of dried blood spot for therapeutic drug monitoring and individualized patient tailor dosing;
- Need of shorter treatment for sensitive TB and MDR-TB, with strongly sterilizing components;
- Use of older drugs like RMP, Pyrazinamide in higher doses in new regimens;
- Clinical case and MDR-TB regimen design according to WHO Guidelines and new anti-TB drugs and regimens;
- Micronutrients (Vitamin A, D) role in MDR-TB the treatment.

## Panel discussion

### All speakers

- The PK and maximum tolerated doses for the first line TB drugs are not well-established;
- Over-the-counter availability of TB drugs needs to be avoided and the regulatory role strengthened;
- Changes in national TB programs are necessary to improve control of the drugs, regulate over the counter prescriptions;
- Acquired resistance versus transmitted resistance (Case of Mozambique, Mobility to South Africa and mining communities);
- Phenotypic versus genotypic resistance testing: which one better correlates with outcome? Both need to be taken in account, studies are still needed;
- Synergism between drugs can only be tested phenotypically;
- Is there a difference in virulence between sensitive TB and drug resistant TB? Drug resistant TB is as bad as sensible TB, there is more difference in strains, Beijing is more virulent, affecting younger people.
